# Inter‐Individual Variation in LUNG Mechanics Assessed by the Non‐Invasive PEEP‐Step Method During Laparoscopic and Robotic Surgery Indicates Need for Individualized Ventilatory Settings

**DOI:** 10.1111/aas.70388

**Published:** 2026-11-05

**Authors:** Ali Abdulhussein, Sara Hammarskjöld, Ola Stenqvist, Per Persson, Katarina Hallén, Pether Jildenstål, Sophie Lindgren

**Affiliations:** ^1^ Sahlgrenska University Hospital Institute of Clinical Sciences Gothenburg Sweden; ^2^ Ryhov County Hospital Futurum Academy for Health and Care Jönköping Sweden; ^3^ Department of Health Sciences Lund University Lund Sweden; ^4^ Faculty of Nursing and Health Sciences Nord University Bodö Norway; ^5^ Department of Anesthesiology and Intensive Care Skane University Hospital Lund Sweden

**Keywords:** head down tilt, laparoscopy, lung volume measurements, PEEP‐step method, pneumoperitoneum, respiratory mechanics, robotic surgical procedures

## Abstract

**Background:**

During laparoscopic surgery, abdominal carbon dioxide (CO_2_) insufflation in the supine position and with head‐down tilt for robotic surgery increases abdominal pressure and pushes the diaphragm cranially. This causes a decrease in end‐expiratory lung volume and in lung and chest wall compliance. These effects can be counteracted by the application of PEEP. Our study aimed to measure lung and chest wall mechanics during laparoscopic surgery using a non‐invasive method (PEEP‐step method) and to determine the optimal PEEP level for each patient.

**Methods:**

Thirty‐two patients scheduled for abdominal surgery participated in this observational study with pneumoperitoneum. Fourteen patients underwent surgery for pelvic conditions in head‐down position and 18 in supine position. Data from the ventilator was collected and analysed with a dedicated software, which calculated lung compliance and transpulmonary pressure from a two‐step PEEP increase. Measurements were performed after induction, after pneumoperitoneum in supine/tilt head‐down and before emergence.

**Results:**

Lung and chest wall compliance decreased during CO_2_ insufflation with high individual variation, and we observed a≈90% increase in transpulmonary driving pressure. After CO_2_‐desufflation, lung and chest wall compliance returned to near baseline levels on a group level. However, 19 patients had a remaining reduction in lung compliance, six with 20%–50%. Optimal PEEP level calculated by the software was twice the clinical routine level.

**Conclusions:**

The study found substantial individual variation in lung compliance during laparoscopic surgery in patients without evident lung disease. This variability does not align well with respiratory system compliance. The findings highlight the need for individualized ventilator settings based on lung‐ rather than respiratory system mechanics. The PEEP‐step method may be a non‐invasive, real‐time bedside tool to identify patients who would benefit from higher PEEP to preserve lung volume and possibly prevent postoperative complications.

**Editorial Comment:**

Laparascopic surgical conditions can affect lung mechanics, though not always predictably. This study examined how a test of higher or lower PEEP directly after anaesthesia induction might inform PEEP optimization in the same cases undergoing laparoscopic surgery.

**Trial Registration:**

ClinicalTrials.gov (NCT04484727, 2022‐05‐01)

## 1 Introduction

Laparoscopic surgery with abdominal carbon dioxide (CO_2_) insufflation, as well as robot assisted surgery for pelvic conditions in steep head‐down tilt, are risk factors for postoperative pulmonary complications (PPC) [1, 2, 3, 4]. Currently, protective ventilation with tidal volumes (V_T_) of 6–8 mL/kg ideal body weight (IBW) is recommended during general anaesthesia, but high versus low PEEP has been tried without clear reduction of PPC incidence [5]. During robot assisted laparoscopic surgery a PEEP of 8 cmH_2_O has been described as a sufficient level to prevent formation of atelectasis [6].

Even in lung healthy patients, that is, patients without a history of lung disease, respiratory mechanics exhibit significant intraindividual variation with respiratory system compliance (C_RS_) of 60 ± 13, corresponding to a 95% confidence interval of 34–86 mL/cmH_2_O [7], which may explain why high versus low PEEP has been evaluated with inconclusive results [5, 8]. Thus, a number of studies [9] have advocated individualization of PEEP according to electric impedance tomography (EIT) measurements [10], oxygenation [11], best C_RS_ [12] and airway driving pressure (ΔP_AW_) [13, 14].

Transpulmonary driving pressure (ΔP_L_) is the pressure that directly affects lung tissue. The ΔP_AW_, which is monitored in clinical practice, is the combined driving pressure of the lung, ΔP_L_, which overcomes the recoil of the lung, and the driving pressure of the chest wall (ΔP_CW_), which pushes away the chest wall complex (ΔP_L_ + ΔP_CW_). C_RS_ is estimated from ΔP_AW_, and lung compliance (C_L_) is estimated from ΔP_L_. Transpulmonary driving pressure varies between 30% and 80% of ΔP_AW_ [15, 16, 17, 18, 19], which means that, for example, in a patient with a ΔP_AW_ of 12 cmH_2_O, ΔP_L_ can vary between 3.6 and 9.7 cmH_2_O, the latter being close to the upper safety limit for transpulmonary driving pressure [15, 18, 19]. Thus, the use of C_RS_ and ΔP_AW_ may not be adequate surrogates to guide selection of tidal volume and PEEP.

Transpulmonary pressure calculation has so far required oesophageal pressure measurements, which are time consuming and therefore less suitable for use in a surgical setting (OR) [11, 20, 21]. We have developed a non‐invasive method, the PEEP‐step method (PSM) for separation of lung and chest wall mechanics, based on the finding that the PEEP induced change in end‐expiratory lung volume (ΔEELV) is determined by the size of the PEEP‐step and lung compliance (ΔPEEP × C_L_) [22] (Figure S9 of e‐supplement). Consequently, if ΔEELV is determined, C_L_ can be calculated as ΔEELV/ΔPEEP. PSM allows rapid determination (< 3 min) of C_L_, transpulmonary driving pressure as well as identification of PEEP level with lowest transpulmonary driving pressure (for physiological background and validation, see e‐supplement) [17, 18, 22, 23, 24, 25, 26, 27].

The focus of this prospective observational study was to assess, using the PSM, the large inter‐individual variation in lung mechanics and response to PEEP during laparoscopic surgery in supine and with tilt head‐down by

1. Determining C_L_, transpulmonary driving pressure and optimal mechanical PEEP, that is, the PEEP level where C_L_ is highest and transpulmonary driving pressure lowest (least injurious) immediately after induction.
2. Describing the effect on lung mechanics of perioperative abdominal CO_2_ insufflation in supine and head‐down tilt.
3. Assessing the degree of recovery of C_L_ before wake‐up as compared to just after induction.

## 2 Methods

This prospective observational study was performed on 32 patients scheduled for laparoscopic surgery with abdominal CO_2_ insufflation, 18 in supine and 14 patients with head‐down tilt of 28° ± 3° for robotic surgery. These patients constitute the first cohort of patients of the total of 200 patients that was approved by the Swedish Ethical Review Authority (decision no. 2020‐01783, 2020‐05‐19) for PSM measurements in the OR (operating room) and ICU (intensive care unit). The main study was registered at ClinicalTrials.gov (NCT04484727, 2022‐05‐01). Patients were recruited from May 2022 to February 2024 in Sahlgrenska University Hospital, Sweden during the preoperative evaluation and after informed consent was apprehended. Inclusion criteria were age > 18, ASA Class I–III. Exclusion criteria were heart failure or advanced lung disease, including chronic obstructive pulmonary disease and emphysema. All patients were anaesthetized and intubated according to department routine procedure, for example, Propolipid, Fentanyl, Rocuronium, Sevoflurane and Remifentanyl, decided by attending anaesthesiologist. The patients were ventilated in volume control ventilation (VCV) with a V_T_ of ≈6–7 mL/kg IBW during the whole anaesthetic procedure, I:E ratio of 1:2, and a respiratory rate of 15–18/min. Base‐line PEEP, 5–9 cmH_2_O was set according to department routine by attending anaesthesiologist and maintained mainly unchanged through the anaesthetic (Tables 1 and 2). The manuscript is prepared in accordance with STROBE [28].

**TABLE 1:** Patient characteristics.

Characteristics	Patients and metrics
Patients assessed (*n*)	44
Patients included (*n*)	34
Patients analysed (*n*)	32
Gender *n* (%)	
Male	15 (47)
Female	17 (53)
ASA‐classification *n* (%)	
I	7 (22)
II	22 (69)
III	3 (9)
Procedure *n* (%)	
Emergency/scheduled	6/26 (19/81)
Conventional laparoscopy	18 (56)
Colorectal	13 (41)
Bowel other	2 (6)
Cholecystic	3 (9)
Robot assisted laparoscopy	14 (44)
Gynaecological	8 (25)
Urological	6 (19)
Age (years)	63 ± 16
Body mass index (kg/m^2^)	28 ± 5
Height (cm)	171 ± 10
Weight (kg)	82 ± 60
Ideal body weight (kg)	65 ± 8
Tidal volume (ml)	425 ± 71
Tidal ventilation by IBW (ml/kg)	7 ± 1
Insufflation pressure (mmHg)	12 ± 2
Angle of head down tilt (°)	28 ± 3

*Note:* ASA, American Society of Anesthesiologists physical status classification system; Mean ± SD.

**TABLE 2:** Baseline PEEP data for all patients and separately for patients in supine or head‐down tilt position at different measuring time points.

	Patients	IND	CO_2_, tilt + CO_2_	Wake‐up
V_T_ (ml)	All (*n* = 32)	426 ± 72	427 ± 71	430 ± 70
	CO_2_ + tilt (*n* = 14)	468 ± 60	466 ± 58	467 ± 52
	CO_2_ (*n* = 18)	394 ± 64	394 ± 64	397 ± 69
PEEP (cmH_2_O)	All	6.7 ± 1.8	6.7 ± 1.9	7.1 ± 2.2
	CO_2_	5.4 ± 0.1	5.3 ± 0.1	5.4 ± 0.1
	CO_2_ + tilt	7.9 ± 1.8	8.7 ± 1.3	9.0 ± 1.8
C_RS_ (ml·cmH_2_O^−1^)	All	62 ± 13	36 ± 10*	59 ± 12
	CO_2_	61 ± 15	40 ± 9*	58 ± 12
	CO_2_ + tilt	65 ± 11	30 ± 7*	60 ± 12
C_L_ (ml·cmH_2_O^−1^)	All	98 ± 27	54 ± 14*	93 ± 26
	CO_2_	91 ± 19	58 ± 13*	83 ± 22
	CO_2_ + tilt	108 ± 34	47 ± 12*	104 ± 27
C_CW_ (ml·cmH_2_O^−1^)	All	180 ± 52	120 ± 57*	189 ± 75
	CO_2_	178 ± 55	141 ± 64*	216 ± 81
	CO_2_ + tilt	183 ± 49	91 ± 28*	158 ± 54
C_loa_ (ml·cmH_2_O^−1^)	All	95 ± 34.8	52 ± 19.7*	89 ± 33
	CO_2_	79 ± 16	50 ± 13*	72 ± 20
	CO_2_ + tilt	117 ± 41	53 ± 27*	107 ± 35
ΔP_AW_ (cmH_2_O)	All	7 ± 1.2	12.6 ± 3.7*	7.5 ± 1.4
	CO_2_	6.7 ± 1.2	10.2 ± 1.8*	7.0 ± 1.3
	CO_2_ + tilt	7.4 ± 1.2	15.9 ± 2.9*	8.0 ± 1.5
ΔP_L_ (cmH_2_O)	All	4.5 ± 0.9	8.4 ± 2.4*	4.9 ± 1.3
	CO_2_	4.4 ± 0.8	7.0 ± 1.4*	5.0 ± 1.3
	CO_2_ + tilt	4.6 ± 1.0	10.3 ± 2.1*	4.8 ± 1.4
Opt PEEP (cmH_2_O)	All	12.8 ± 2.7	NA	NA
	CO_2_	12.0 ± 2.0	NA	NA
	CO_2_ + tilt	14.3 ± 2.6	NA	NA
ΔP_L_ opt PEEP (cmH_2_O)	All	3.1 ± 0.7	NA	NA
	CO_2_	3.0 ± 0.6	NA	NA
	CO_2_ + tilt	3.2 ± 0.8	NA	NA
P_Lplat_ opt PEEP (cmH_2_O)	All	15.9 ± 2.9	NA	NA
	CO_2_	14.6 ± 2.5	NA	NA
	CO_2_ + tilt	17.5 ± 2.6	NA	NA
ΔEELV at opt PEEP (ml)	All	629 ± 225	NA	NA
	CO_2_	638 ± 212	NA	NA
	CO_2_ + tilt	618 ± 249	NA	NA
SpO_2_ (%)	All	98 ± 1	98 ± 2	98 ± 2
	CO_2_	98 ± 2	98 ± 1	98 ± 2
	CO_2_ + tilt	98 ± 1	99 ± 2	99 ± 2
FiO_2_ (%)	All	32 ± 5	31 ± 2	32 ± 4
	CO_2_	33 ± 6	31 ± 3	32 ± 4
	CO_2_ + tilt	30 ± 2	31 ± 2	33 ± 4
MAP (mmHg)	All	73 ± 13	84 ± 9*	71 ± 11
	CO_2_	68 ± 10	79 ± 8*	68 ± 9
	CO_2_ + tilt	80 ± 14	90 ± 7*	75 ± 11

*Note:* * = IND vs CO_2_/CO_2_ + tilt, *p* < 0.05.

Abbreviations: CCW, chest wall compliance; CL, lung compliance; CLoa, overall lung compliance = (ΔEELV_1_ + ΔEELV_2_ + VT)/(P_LplatPEEP2_–PEEP_BL_); CRS, respiratory system compliance; FiO₂, inspiratory oxygen; MAP, mean arterial pressure; NA, Non‐Applicable; Opt PEEP, PEEP level with lowest transpulmonary driving pressure; PEEP, positive end‐expiratory airway pressure; P_Lplat_ opt PEEP, Transpulmonary plateau pressure at optimal PEEP; SpO₂, oxygen saturation by pulse oximetry; VT, Tidal volume; ΔPAW, airway driving pressure; ΔP_L_, transpulmonary driving pressure; ΔP_L_ opt PEEP, Transpulmonary driving pressure at optimal PEEP level; ΔEELV at opt PEEP, end‐expiratory lung volume increase at optimal PEEP level.

### 2.1 Measurement Procedure and Calculation of Lung P/V Curve

The PSM software (Dencker Developments, Lund, Sweden) guided the researcher through the measurement procedure: (1) ‘Await steady state’. (2) ‘Increase PEEP to x cmH_2_O’ (PEEP_1_). (3) ‘Increase PEEP to y cmH_2_O’ (PEEP_2_). (4) *‘*Return PEEP to baseline level’. PEEP was increased by a default value of 70% of ΔP_AW_ at baseline PEEP, as ΔP_L_ is on average 70% of ΔP_AW_ [16, 29, 30, 31], and ΔP_L_ of a tidal volume which is equal to ΔEELV is equal to ΔPEEP [22]. PEEP_1_ was maintained for 1.5 min and PEEP_2_ for ≈1 min. The PSM software accessed data of inspiratory and expiratory tidal volume, inspiratory plateau pressure and end‐expiratory airway pressure, breath‐by‐breath. ΔEELV was calculated as the cumulative difference in inspiratory and expiratory tidal volume between PEEP levels [25, 32] (for details, see e‐supplement, Figures S10 and S11). Calculations of the lung pressure/volume (P/V) curve and optimal PEEP are described in Figures 1, S11—S13 of e‐supplement. Optimal PEEP provided by the PSM software (Figure S14 of e‐supplement) was not used to guide the ventilatory care of the patients. Overall lung compliance (C_Loa_) was calculated as the volume between end‐expiration at baseline PEEP and the end‐inspiratory volume at PEEP_2_ divided by the end‐inspiratory transpulmonary plateau pressure at PEEP_2_ minus baseline PEEP (ΔV_oa_/ΔP_Loa_, where ΔV_oa_ = ΔEELV_1_ + ΔEELV_2_ + V_T_ and ΔP_Loa_ = P_LplatPEEP2_ − PEEP_BL_) (Figures 1G and S11G).

**FIGURE 1:**
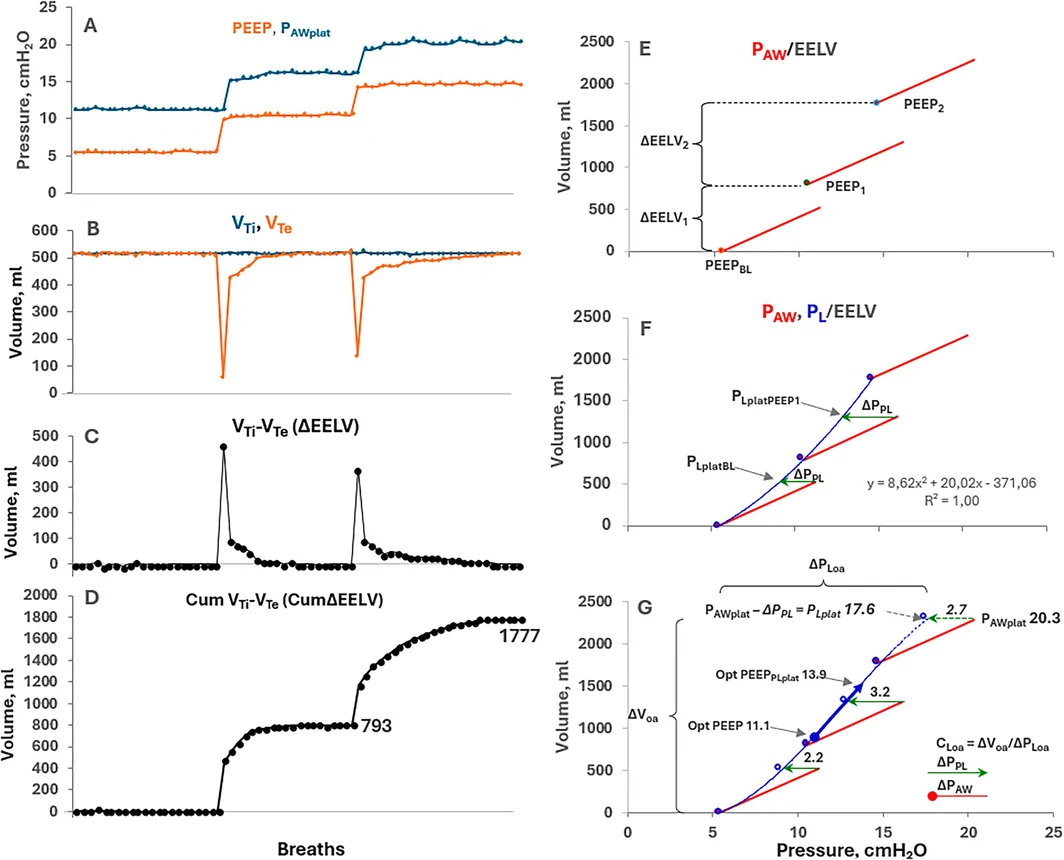
The two PEEP‐step measurement procedure and calculations. **Panel A:** PEEP and airway plateau pressure. **Panel B:** Inspiratory and expiratory tidal volume (V_Ti_, V_Te_), where the offset between V_Ti_ and V_Te_ at steady state has been corrected for at baseline, at PEEP_1_ and at PEEP_2_ (for details, see e‐supplement). **Panel C:** breath‐by‐breath difference between V_Ti_ and V_Te_ (ΔEELV). **Panel D:** Cumulative ΔEELV calculated as the cumulative difference between V_Ti_ and V_Te_ between PEEP levels. **Panel E:** Tidal airway P/V curves positioned at respective PEEP/ΔEELV levels (PEEP_BL_, PEEP_1_, PEEP_2_, ΔEELV_1_ = volume PEEP_BL_−PEEP_1_, ΔEELV_2_ = volume PEEP_1_−PEEP_2_) [25, 32]. **Panel F:** An equation of the best‐fit lung P/V curve connecting the end‐expiratory airway = transpulmonary pressure P/V points was calculated. The green arrows indicate pleural driving pressure (ΔP_PL_) and point at the transpulmonary plateau pressure (P_Lplat_) of baseline PEEP and PEEP_1_ on the best‐fit lung P/V curve. These transpulmonary plateau pressures were determined by calculating the volume at steps of 0.1 cmH_2_O from the equation for the best‐fit curve to find the pressure where the calculated volume was equal to the end‐inspiratory airway plateau pressure at baseline PEEP and PEEP_1_ (Figure S12). **Panel G:** The complete lung P/V curve calculated from baseline PEEP to end‐inspiratory transpulmonary plateau pressure at PEEP_2_, which was estimated as the airway plateau pressure at PEEP_2_ minus the average of ΔP_PL_ at PEEP_BL_ and PEEP_1_. Estimated values in italics and dashed lines. Blue arrow: Tidal lung P/V curve at optimal PEEP level half a tidal volume below the steepest P/V point of the lung P/V curve [7, 10] (for details see e‐supplement, Figure S13). Overall lung compliance (C_Loa_) was calculated as ΔV_oa_/ΔP_Loa_, where ΔV_oa_ = ΔEELV_1_ + ΔEELV_2_ + V_T_ and ΔP_Loa_ = P_LplatPEEP2_−PEEP_BL_.

### 2.2 Statistics

The present study is observational with focus on inter‐individual variations in lung mechanics. As there is no previous data on C_L_, ΔP_L_ and EELV changes in response to pneumoperitoneum and PEEP with or without head‐down tilt, sample size calculation was not possible. However, post hoc sample size analysis was performed using absolute values of C_L_ and percentage decrease in C_L_ in the two groups of CO_2_ and CO_2_ + tilt. A power of 70% using an alpha value of 0.05 was found for the absolute C_L_ values and 100% for the percentage difference between groups (ClinCalc LLC 2026. All rights reserved. https://clincalc.com/Stats/SampleSize.aspx, North Chicago, IL, USA), which indicates that the groups were sufficiently large for statistical comparisons.

Differences in transpulmonary pressure, driving pressure, C_L_, C_RS_, and lung volumes as well as PEEP values were normally distributed as assessed by histogram and Shapiro–Wilk test (Statistics Kingdom 2017, Fit tests calculator, https://www.statskingdom.com/shapiro‐wilk‐test‐calculator.html, Melbourne, Australia). Distributional data are presented as mean + standard deviation and range. Paired comparisons between the predefined time points were performed using Students *t*‐test. Unpaired *t*‐tests were used to analyse differences in percentage reduction of C_L_ and C_Loa_ between groups CO_2_ and CO_2_‐tilt. A *p*‐value of < 0.05 was considered significant. (Excel, Microsoft Office 365) Bonferroni correction was used for multiple pairwise comparisons.

## 3 Results

### 3.1 Base‐Line Data

This study included 32 adult ASA I–III patients scheduled for elective or acute urological (*n* = 6), gynaecological (*n* = 8) or colorectal/bowel/cholecystic (*n* = 18) laparoscopic surgery with abdominal CO_2_ insufflation (Table 1).

### 3.2 Lung Compliance, Transpulmonary Driving Pressure and Optimal PEEP After Induction

A total of 44 patients were assessed. Ten patients were excluded as they met the exclusion criteria or due to alterations in operating schedules. Two of the included patients were lost to follow‐up due to software failure. Data from 32 patients were included in the analyses; however, data from three measure points were not interpretable. Pneumoperitoneum was induced by inflation of CO_2_ to a pressure of 12 ± 2.1 mmHg (≈16 ± 2.1 cmH_2_O). Patients were positioned supine (*n* = 18) or with a 28 ± 3‐degree head‐down tilt (*n* = 14) during the procedure. During measurement, oxygen saturation (SpO_2_) and mean arterial pressure (MAP) were minimally affected, and no additional circulatory support was required. FiO_2_ could be kept around 30% in all patients (Table 2). During the two‐PEEP‐step measurement procedure from baseline PEEP≈7, to 12 and finally 17 cmH_2_O, the lung P/V curve from baseline PEEP to P_Lplat_ at highest PEEP level was automatically calculated by the PSM software (Figures 2 and S8 of e‐supplement). C_L_ at baseline PEEP was 98 ± 27, (57–203) ml/cmH_2_O. Corresponding C_RS_ was 62 ± 13, (37–90) ml/cmH_2_O (Table 2). C_L_ increased more than 30%, to 131 ± 32 mL/cmH_2_O, between baseline PEEP and PEEP_1_ during the measurement procedure after induction. At the highest PEEP level, C_L_ decreased to 106 ± 36 mL/cmH_2_O (Figure S1, e‐supplement). The response of airway and transpulmonary driving pressure to the two PEEP steps showed that ΔP_AW_ and ΔP_L_ could respond differently, even in opposite directions (Figure 3).

**FIGURE 2:**
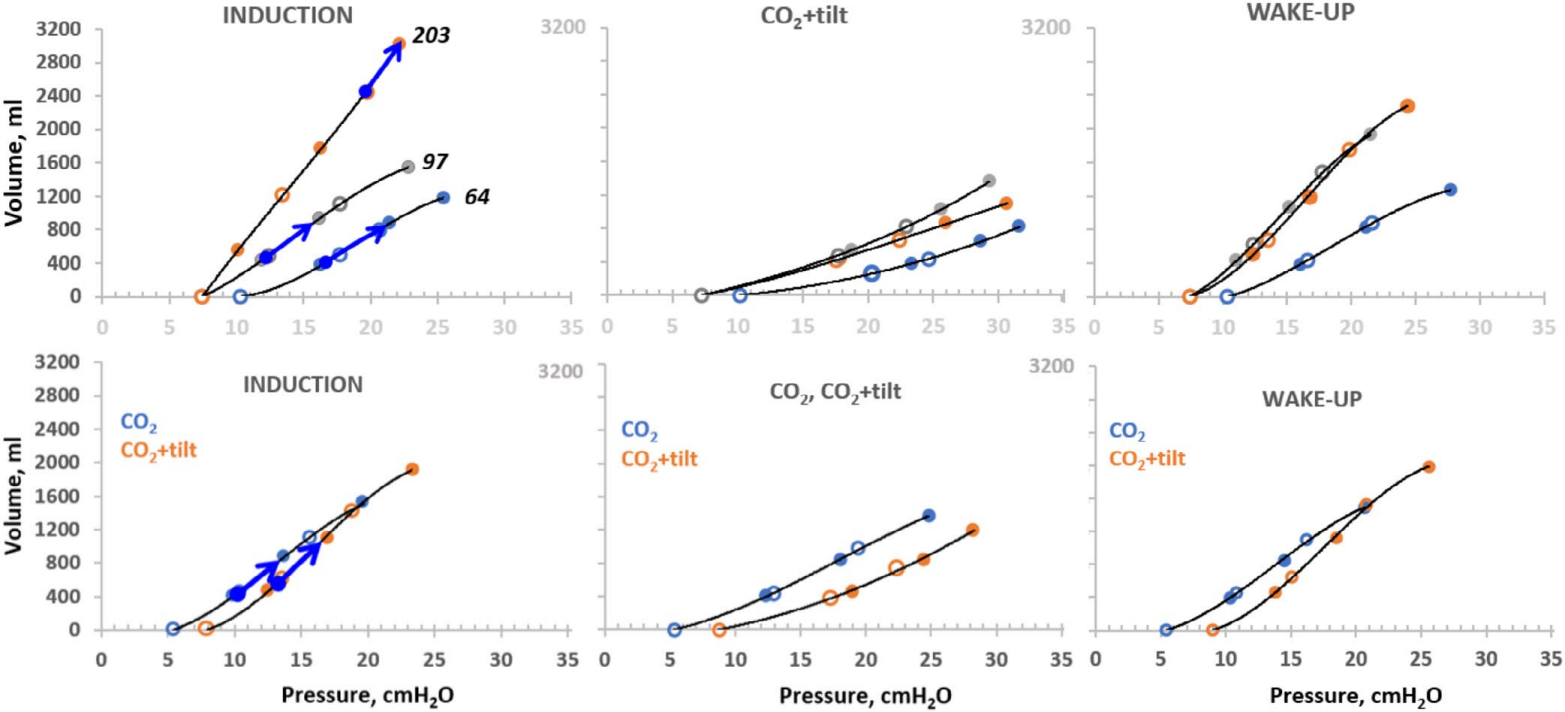
Effect of induction, CO_2_‐insufflation and position on lung mechanics and optimal PEEP displayed as: Lung P/V curves after induction (**left panels**), after CO_2_ and CO_2_ + tilt (**mid panels**), and before wake‐up (**right panels**). Optimal PEEP determined after induction (blue arrows). **Upper panels** show individual lung P/V curves during the three measurement points for the CO_2_ + tilt patients with lowest C_Loa_, 64 (blue dots), median 97 (grey dots) and highest C_Loa_, 203 mL/cmH_2_O (brown dots). **Lower panels** show mean lung P/V curves during the three measurement points for the patients with CO_2_ (blue dots) in supine and CO_2_ + tilt (brown dots). Filled dots: end‐inspiratory P/V points, open circles: end‐expiratory P/V points.

**FIGURE 3:**
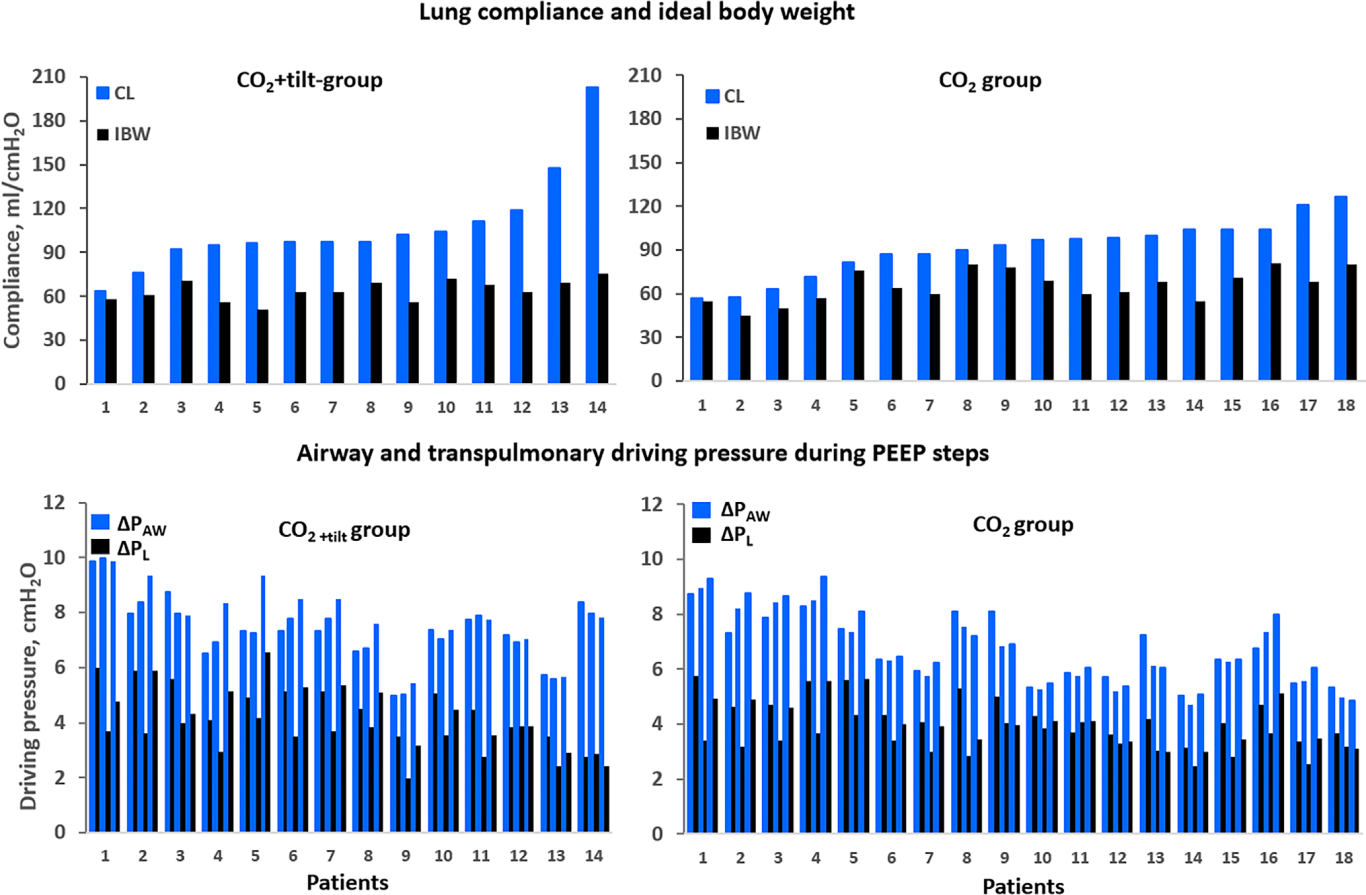
Ideal body weight (IBW) does not reflect the individual elastic properties of the lung and individual changes in airway driving pressure (ΔP_AW_) during PEEP changes, do not coincide with changes in transpulmonary driving pressure (ΔP_L_). **Upper panels:** Inter‐individual variation of lung compliance (C_L_) (blue bars) and IBW (black bars) with patients ranked according to C_L_ at baseline PEEP after induction. **Lower panels**: ΔP_L_ (black bars) and ΔP_AW_ (blue bars) at baseline PEEP, PEEP_1_ and PEEP_2_ of the measurement procedure. **Left panels:** robot‐assisted surgery (CO_2_+ tilt group). **Right panels:** conventional laparoscopic surgery (CO_2_ group).

### 3.3 Optimal PEEP

Optimal PEEP level was 12.8 ± 2.7 cmH_2_O with an increase in EELV over baseline PEEP of 629 ± 225 mL. ΔP_L_ at optimal PEEP was 3.1 ± 0.7 cmH_2_O, 30% lower than at baseline PEEP. Transpulmonary plateau pressure at optimal PEEP was 15.9 ± 2.9 cmH_2_O (Table 2, Figures S2–S6,S8 of e‐supplement).

### 3.4 Effect on Lung Mechanics of Abdominal CO_2_ Insufflation in Supine and Head‐Down Tilt

Both CO_2_‐insufflation in supine and CO_2_‐insufflation + tilt caused a significant decrease in chest wall compliance (C_CW_) with 20 ± 28 and 51% ± 23% respectively, *p* < 0.05. Corresponding C_L_ decrease was 35% ± 11% and 55% ± 10%, *p* < 0.05 (Figure 4).

**FIGURE 4:**
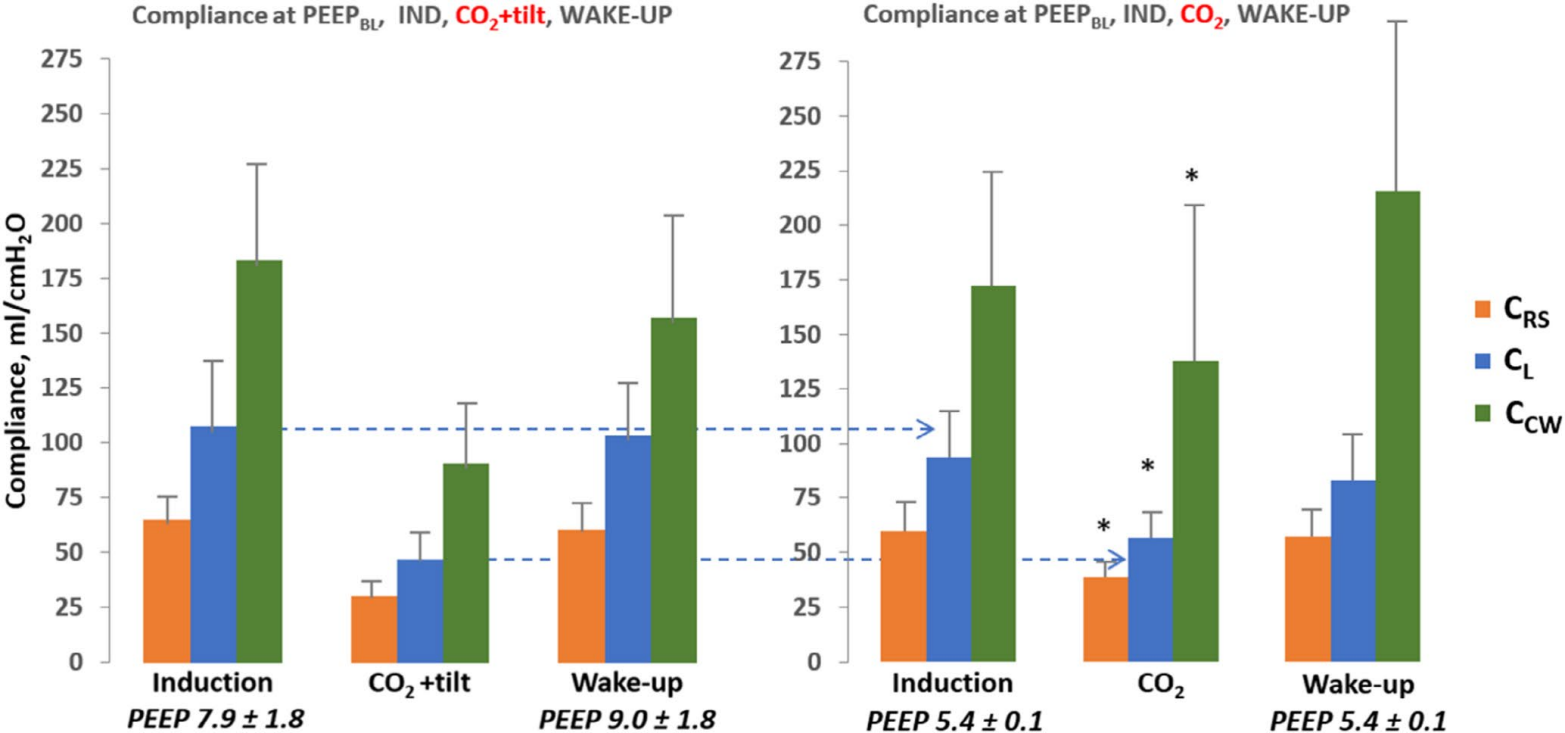
Abdominal CO_2_ insufflation during supine position for conventional laparoscopy (*n* = 18) or in head‐down tilt position during robot‐assisted surgery (*n* = 14), reduces lung compliance (C_L_) as well as chest wall compliance (C_CW_). Blue dashed arrows indicate that lung compliance is higher in the CO_2_ + tilt group at baseline after induction but decreases more during CO_2_ + tilt than in the patients with CO_2_ in supine. **Left panel:** respiratory system compliance (C_RS_), C_L_ and C_CW_ at baseline PEEP after induction (IND), during CO_2_ insufflation + tilt head‐down 28 ± 3 degrees (CO_2_ + tilt) and before emergence (wake‐up). **Right panel:** effects of CO_2_‐insufflation in supine (CO_2_). * Indicate significant effect of CO_2_ insufflation and tilt, *p* < 0.05.

The response in C_L_ and ΔP_L_ at baseline PEEP to CO_2_ and CO_2_‐tilt displayed a considerable variation. In individual patients, ΔP_L_ reached close to and above the safety level of 9–10 cmH_2_O in the CO_2_‐tilt group (Figures 5, 6, S7 of e‐supplement).

**FIGURE 5:**
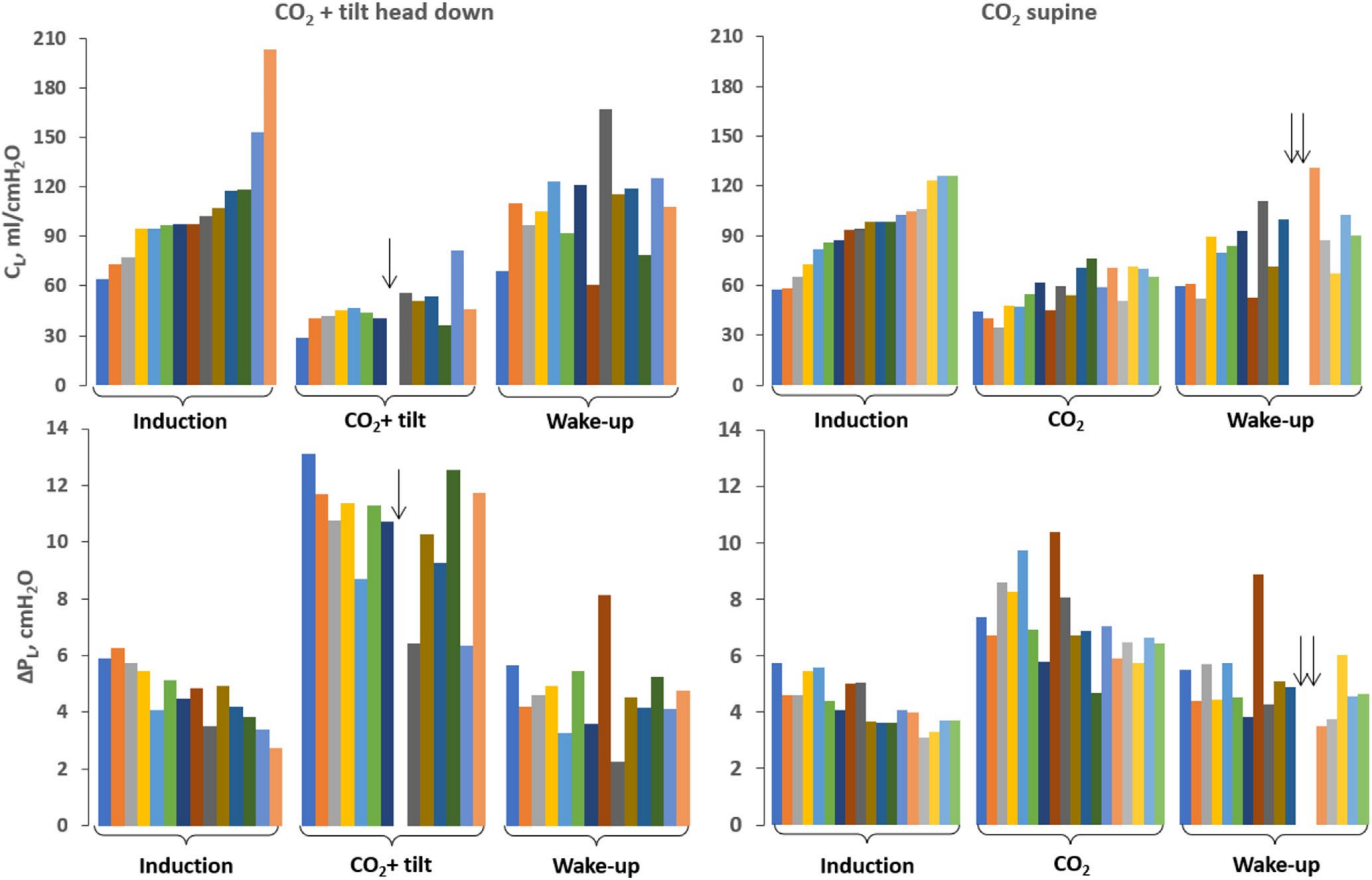
Considerable individual variation in lung compliance (C_L_) and transpulmonary driving pressure (ΔP_L_) in response to CO_2_ insufflation and head tilt down were observed during conventional laparoscopic surgery in supine (*n* = 18) and/or during robot‐assisted surgery (*n* = 14). Measurements were performed at baseline PEEP after induction, after CO_2_ insufflation and/or tilt head down (CO_2_ and CO_2_ + tilt) and before emergence (wake‐up). **Left panels:** robot‐assisted surgery (CO_2_ + tilt head down) **Right panels:** conventional laparoscopic surgery (CO_2_). Black vertical arrows indicate missing data. Patients are ranked by C_L_ after induction (upper left panel, induction).

**FIGURE 6:**
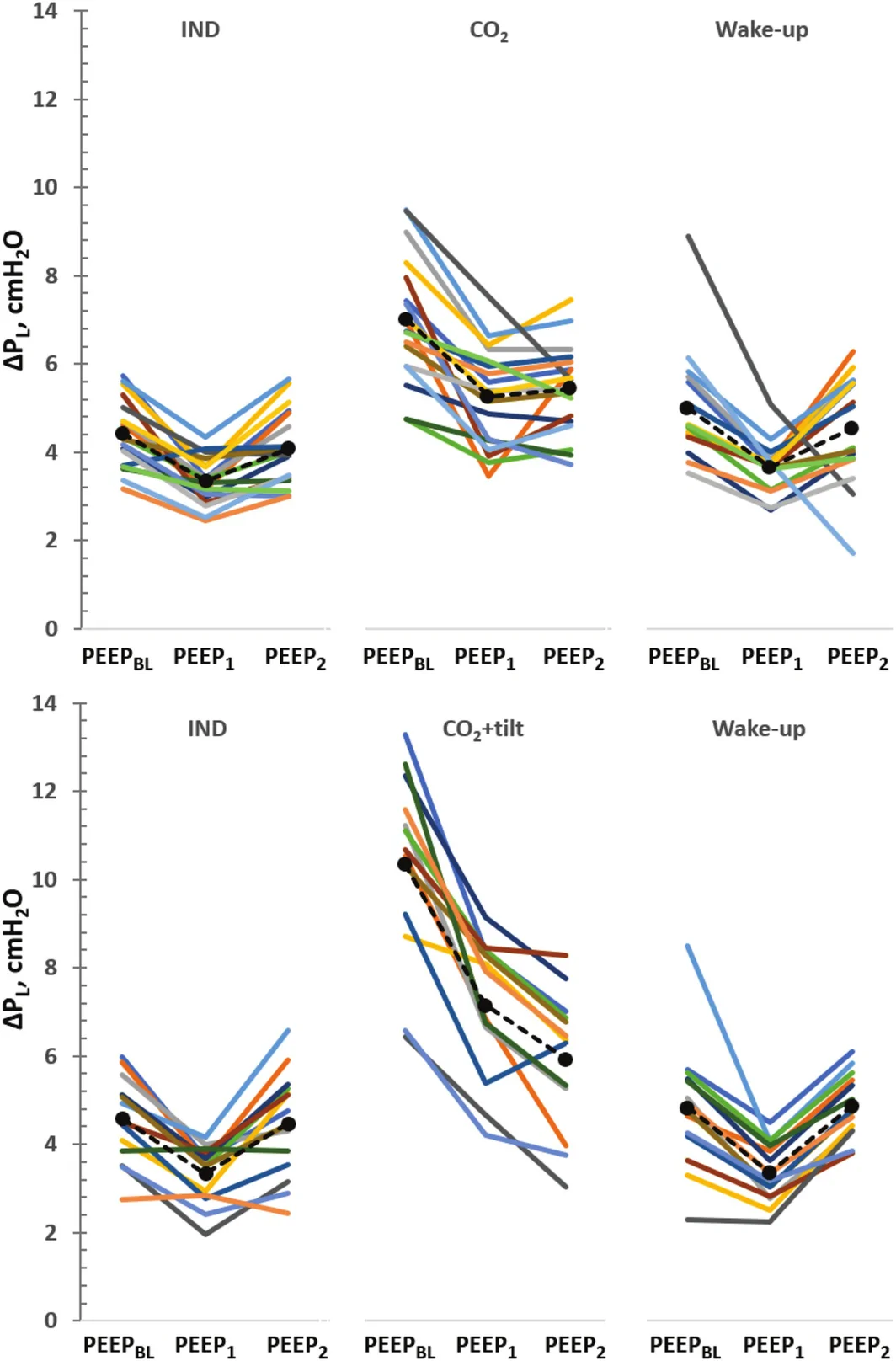
Observations of marked individual variations in transpulmonary driving pressure (ΔP_L_) during the stepwise PEEP increase of the measurement procedure, especially after CO_2_‐insufflation and head‐down tilt in the robot‐assisted surgery group (lower mid panel). Measurements were performed after induction (IND, **left panels**), after CO_2_ insufflation or CO_2_‐insufflation and head‐down tilt (**mid panels**) and before wake‐up (**right panels**). The black dashed line indicates mean values of ΔP_L_. **Upper panels:** individual ΔP_L_ at baseline PEEP (PEEPBL), first PEEP step (PEEP_1_) and second PEEP step (PEEP_2_) in the laparoscopy in supine position group (CO_2_). **Lower panels:** corresponding ΔP_L_ in robot‐assisted surgery group (CO_2_‐tilt).

When increasing PEEP stepwise during the measurement procedure, there was a marked variation in response of ΔP_L_ at all three measurement occasions, especially during CO_2_ and CO_2_ + tilt. After CO_2_ insufflation in supine, the increase in ΔP_L_ between PEEP_1_ and PEEP_2_ was minimal, whereas after CO_2_‐tilt, ΔP_L_ continued to decrease at PEEP_2_. In contrast, during measurements after induction and before emergence, ΔP_L_ increased at PEEP_2_. In individual patients, ΔP_L_ reached close to and above the safety level of 9–10 cmH_2_O in the CO_2_‐tilt group (Figure 6).

### 3.5 Recovery of Lung Compliance Before Wake‐Up

Overall C_L_ in the CO_2_‐supine patients, C_Loa_, decreased 36% ± 14% between induction and CO_2_‐insufflation compared to 58% ± 19% in the CO_2_‐tilt group. Before emergence, C_Loa_ had recovered 92% ± 16%, range 68%–117% in the CO_2_ group and to 94% ± 25%, range 50%–146% in the CO_2_‐tilt group compared to CL_oa_ at induction (Figure 7).

**FIGURE 7:**
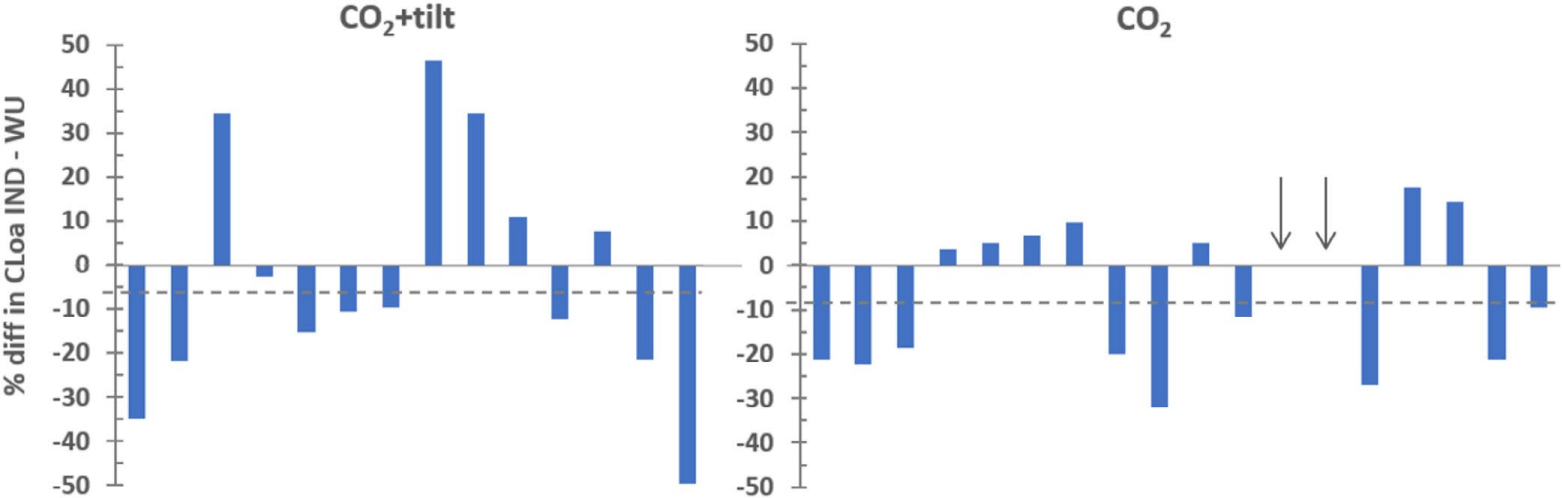
We observed a large individual variation in recovery of lung compliance before emergence and many patients did not return to baseline, which is described as the difference (%) in overall lung compliance (C_Loa_) before wake‐up (WU) compared to C_Loa_ baseline after induction (IND). **To the left** 14 robot‐assisted surgery patients (CO_2_ + tilt) and **to the right** 18 conventional laparoscopy patients (CO_2_). The grey dashed line shows the limited average decline in C_Loa_ of 8% and 6% in the CO_2_ and CO_2_ + tilt group, respectively. C_Loa_ is C_L_ calculated from total volume and pressure changes during the whole two‐PEEP‐step manoeuver. Black vertical arrows indicate missing data points.

## 4 Discussion

In patients without overt lung disease, we found a large inter‐individual variation in C_L_, ranging from 57 to 203 mL/cmH_2_O. PEEP‐induced changes in C_RS_ did not reflect changes in C_L_ on an individual basis. IBW did not correspond to C_L_ and consequently, tidal volume selection based on IBW does not correspond to individual patient C_L_. Transpulmonary driving pressure was generally low at baseline after induction. Insufflation of CO_2_ alone or in combination with head‐down position caused a significant decrease, not only in C_CW_, but also C_L_. Consequently, transpulmonary driving pressure increased markedly. Before emergence, overall C_L_ had recovered on average just over 90%, but in individual patients, recovery was as low as 50%.

### 4.1 Methodological Considerations

The PEEP‐step procedure could be performed without any training as the software guided the user through the procedure, as described in methods section. Since the procedure is short, ≈3 min, the encroachment on OR flow was limited and well tolerated by surgeons. The measurement procedure had minimal effect on MAP, possibly explained by the short increase in PEEP, which causes compensatory vasoconstriction to minimize the reduction in perfusion pressure. This is described in studies on recruitment manoeuvres with short substantial pressure increase, where arterial pressure decreases less than half of the percentage decrease in cardiac output [33, 34]. In addition, the short duration of the highest PEEP‐level, PEEP_2_ (≈1 min), circulatory stable patients and the increase in preload caused by head‐down tilt also contribute. If circulatory instability is anticipated, the increase in PEEP could be lower than the default recommendation increase, without jeopardizing measurements.

### 4.2 Lung Mechanics After Induction

To reduce PPC, a low tidal volume, 6 mL/kg IBW, has been advocated during anaesthesia [35]. However, the present study in patients without a history of lung disease, C_L_, is highly varying, ranging from 57 to 203 mL/cmH_2_O, which is in accordance with reports of C_L_ in lung healthy patients of 108 ± 20 and 102 ± 25 mL/cmH_2_O [18, 30]. Consequently, a tidal volume of 6 mL/kg IBW cannot fit all patients (Figure 2). In the ARDSnet study, where a V_T_ of 6 mL/kg IBW was proposed, mean IBW was 70 kg, with an average V_T_ of 420 mL. In lung healthy with a C_L_ around 100 mL/cmH_2_O this would result in a ΔP_L_ of 4.2 cmH_2_O. As average ΔP_L_/ΔP_AW_ is 0.60–0.70 [25, 32, 36, 37, 38, 39] the upper safety limit for transpulmonary driving pressure is 9–10 cmH_2_O, that is, 60%–70% of the upper safety limit for P_AW_, 15 cmH_2_O [40]. In the present study ΔP_L_ at baseline PEEP was 4.5 ± 0.9, ranging from 2.7 to 6.0 cmH_2_O, well below injurious transpulmonary driving pressure levels.

Low versus high PEEP has been tried without conclusive results [41], possibly as neither a low nor a high PEEP fits all patients due to the great individual variations in lung mechanics and the varying response to PEEP increase (Figures 2, 4, 5). Findings also indicate that C_RS_ is not an adequate surrogate for C_L_ as ΔP_L_ and ΔP_AW_ may change in opposite directions when increasing PEEP (Figure 3) [18].

### 4.3 Effects of Pneumoperitoneum and Head‐Down Tilt Position on Lung Mechanics

CO_2_‐insufflation and head‐down tilt cause decrease in both C_CW_ and C_L_ as due to reduction of EELV [42]. CO_2_‐insufflation causes a cranial push of the diaphragm that affects mostly ventral (non‐dependent) lung. During tilt, gas will initially affect the diaphragm analogously to supine, but when the position is changed to head‐down, gas moves towards the pelvic region. Consequently, the cranial push of gas on the diaphragm is limited. Instead, the weight of the abdominal organs will push on the dorsal part of the diaphragm, reducing EELV and consequently C_L_. Reduction of C_L_ at baseline PEEP is significantly more pronounced in head‐down tilt position than in supine, 58% ± 19% compared to 36% ± 14%. Presumably this is a result of the head‐down tilt causing greater reduction in EELV than CO_2_‐insufflation in supine position. There was a marked increase in ΔP_L_ during CO_2_‐insufflation in both positions (Figure 5). In the CO_2_ + tilt patients, ΔP_L_ exceeded the safety limit of 9–10 cmH_2_O as also reported in another study during CO_2_‐tilt head‐down [43]. However, the high ΔP_L_ of the present study is probably not a result of overstretching the alveoli, but rather a result of decreased lung volume. When increasing PEEP stepwise during the measurement procedure, EELV increases, resulting in a stepwise increase in C_L_ while a decrease in ΔP_L_ was seen, which contradicts overinflation (Table 2, Figures S1, S3, S5 of e‐supplement).

### 4.4 Optimal PEEP

In contrast to standard PEEP titration to find the PEEP level with highest C_RS_ (= lowest ΔP_AW_), which requires 5–8 PEEP steps of 2–5 min duration each, PSM not only determines ΔP_L_, but also ΔEELV. This enables determination of the lung P/V curves by a two PEEP‐step procedure. As end‐expiratory and end‐inspiratory lung P/V points are aligned on a single lung P/V curve, optimal PEEP, the PEEP level where ΔP_L_ is lowest and thus least injurious, can be calculated. At optimal PEEP, ≈13 cmH_2_O, which was almost twice the used clinical PEEP, ΔP_L_ was 30% lower, ≈3 cmH_2_O, than at baseline PEEP. This is lower than ΔP_L_ seen in lung healthy patients [18, 30]. P_Lplat_ was ≈16 cmH_2_O, which is well below the upper safety limit of 25 cmH_2_O [44], indicating that the risk of overdistension should be minimal (Figures 8, S5, S8 of e‐supplement).

**FIGURE 8:**
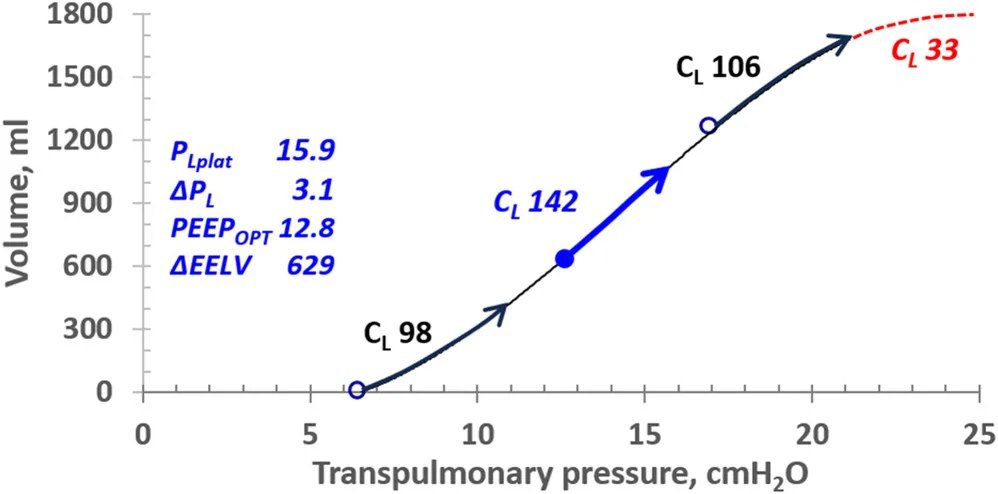
Lung P/V curve of mean data after induction from all 32 patients (thin black line), extrapolated to 25 cmH_2_O (dashed red line with CL value in red). Thick black arrows indicate tidal lung P/V curves at baseline PEEP and PEEP_2_ of the measurement procedure with corresponding lung compliance values. Thick blue arrow depicts tidal lung P/V curve at optimal PEEP. Blue text shows data for the optimal PEEP tidal volume. Note that, while the tidal lung P/V curve at baseline PEEP shows a continuously increasing compliance (upwards concave curve), the corresponding curve at PEEP_2_ shows a continuously decreasing compliance (upwards convex curve), which indicates beginning of overdistension. Lung compliance of the extrapolated lung P/V curve is less than half of lung compliance at optimal PEEP. The curve is close to horizontal when reaching 25 cmH_2_O, which is an indication that the capacity of the lung is reached, where the risk of lung tissue injury is high.

In present study, optimal PEEP was not applied, hence we do not know what impact such a PEEP level would have on circulation. In a study during laparoscopic surgery where optimal PEEP of 12 cmH_2_O was implemented, ‘no circulatory depression or need for increase in fluid or vasoactive drugs was seen’ [10] and in a EIT study, optimal PEEP of 14–18 cmH_2_O was applied without signs of circulatory depression [45]. Despite the relatively high PEEP‐levels used, global values of lung stress and strain remained within physiological limits. In a study on high (≈12 cmH_2_O) versus low PEEP in septic patients, hemodynamic parameters were similar or improved in the high PEEP groups [46].

EELV would have been on average 600 mL higher if the optimal PEEP level had been used (Figure S6, e‐supplement). Focus has been on postoperative complications being a result of injurious driving pressure, [47, 48, 49, 50, 51, 52, 53] but, in the present study, ΔP_L_ is normal before emergence. This indicates that the decrease in EELV and possible atelectasis during laparoscopic surgery may be the cause of PPC, as spontaneous breathing effort will increase with lowered lung volume (law of Laplace).

### 4.5 Recovery of C_L_ Before Extubation

In a study with abdominal CO_2_‐insufflation to a pressure of 16 cmH_2_O in supine, ΔP_AW_ was increased by 18% before wake‐up, compared to after induction [7]. In another study of robotic surgery, ΔP_L_ at extubation was increased by 37% compared to induction [43]. These findings indicate that after CO_2_‐deflation, the lung does not return to baseline lung volume and elastic properties. In the present study C_LOA_ returned to ≈90% of the mean value after induction, but with a great individual variation, ranging from 50% to 146% recovery (Figure 7). Identifying patients who have a large decrease in C_L_ before emergence enables clinicians to consider a recruitment manoeuvre before extubation and/or continuous positive airway pressure (CPAP) postoperatively and thereby possibly avoid PPC.

### 4.6 Limitations

The main limitation of the present study is that the ethical approval did not allow implementation of optimal PEEP to reduce or prevent decrease in EELV. Thus, there is a need for future studies comparing reduction and recovery in C_L_ in patients with standard PEEP and optimal PEEP. Furthermore, follow‐up of oxygenation or other signs of postoperative complications was not performed. The two sub‐groups, CO_2_ and CO_2_‐tilt, were small, but post hoc sample size analysis using absolute and percentage decrease in C_L_ indicates that the groups were large enough for statistical comparison.

## 5 Conclusion

This study demonstrates considerable individual variability in C_L_ in response to abdominal CO_2_ insufflation in supine and head‐down tilt among patients without overt lung disease during conventional laparoscopic and robot‐assisted surgery. As C_RS_ did not reflect C_L_, these findings support individualized ventilatory strategies including tailored tidal volume and PEEP settings based on lung mechanics, rather than respiratory system mechanics. The PEEP step method offers a bedside approach to assess lung mechanics, optimal PEEP and identification of patients in need of recruitment manoeuvres before emergence or CPAP treatment postoperatively, with little depression of arterial pressure when PEEP is increased in two steps during the measurement procedure in a routine clinical setting such as the operating room.

## Author Contributions

Conceived the idea: O.S., P.P. Study concept and design: O.S., S.L. Data acquisition: A.A., S.H., S.L. Data analysis and extraction: A.A., S.H., O.S. Drafting of initial manuscript: O.S., S.L. Critical revisions of the manuscript: All authors. Supervision of the project: S.L., P.J., K.H., O.S. The corresponding author attests that all author contributions meet the criteria for authorship as outlined by the ICMJE guidelines (https://www.ICMJE.org) and that no others have been omitted.

## Funding

This study was supported by ALF grants from the Swedish Research Council (ALFGBG‐965057, ALFGBG‐1005268).

## Disclosure

Preliminary results from this study are published as two abstracts in the European Journal of Anesthesiology Volume 42|e‐Supplement 63|June 2025, 576–577.

## Conflicts of Interest

O.S. holds shares in the Lung Barometry AB (LBAB), which owns the commercial rights to the PSM technology. LBAB has recently entered into an agreement to commercialize PSM with a Med Tech company. All other authors declare no conflicts of interest.

## Supporting information


**Figure S1:** Lung compliance for the patients at baseline PEEP, PEEP_1_ and PEEP_2_ during the measurement procedure after induction (left panel), after CO_2_/CO_2_ + tilt (mid panel) and before wake‐up (right panel). The black dashed line indicates mean values of C_L_.
**Figure S2:** Optimal PEEP calculated by equation for lung P/V curve at induction.
**Figure S3:** Lung compliance at optimal PEEP compared to lung compliance at baseline PEEP at induction.
**Figure S4:** Transpulmonary driving pressure at baseline PEEP (blue bars) and at optimal PEEP (brown bars) at induction.
**Figure S5:** Optimal PEEP, transpulmonary driving pressure and transpulmonary plateau pressure at optimal PEEP. Blue, black and brown dashed lines indicate mean optimal PEEP, ΔP_L_ and P_Lplat_ of optimal PEEP. Red thin dashed line indicate upper safety level of transpulmonary plateau pressure [1] and red thick dashed line indicates upper safety level for transpulmonary driving pressure [2–4].
**Figure S6:** ΔEELV at optimal PEEP after induction. Patients are ranked after C_L_ at baseline PEEP.
**Figure S7:** Upper panels: C_L_ at baseline PEEP at induction, CO_2_ + tilt (left panel) and CO_2_ supine (right panel) and at wake up. Lower panels: ΔP_L_ at baseline PEEP at induction, CO_2_ + tilt (left panel) and CO_2_ supine (right panel) and at wake up. Transpulmonary driving pressure was generally normal‐low at induction.
**Figure S8:** Lung P/V curves of each fourth patients ranked after C_L_ at baseline PEEP direct after induction. Note that the response to a PEEP step increase is highly variable. Overall lung compliance (C_Loa_) is the two‐point compliance between end‐inspiratory lung volume at PEEP_2_ divided by end‐inspiratory transpulmonary plateau pressure at PEEP_2_ minus PEEP at baseline. The thick blue arrow is the tidal lung P/V curve at optimal PEEP, that is, the PEEP level where the transpulmonary driving pressure is lowest (least injurious).
**Figure S9: Left panel:** Correlation plot of cumulative ΔEELV measured PEEP step by PEEP step by the ventilator pneumotachograph versus cumulative ΔEELV calculated PEEP step by PEEP step as ΔPEEP × C_L_ (E_L_ = 1/C_L_) in Katz I [6], Katz II [7], Falke [8], Persson [9], Lundin [10], Stenqvist [11], Garnero [12], Pelosi [13], Gattinoni [14]. E_L_ (= 1/C_L_) in these studies ranged from 9 to 43 cmH_2_O/L (C_L_: 111–23 mL/cmH_2_O) and the ratio of lung elastance to respiratory system elastance (E_RS_ = 1/C_RS_), E_L_/E_RS_ = ΔP_L_/ΔP_AW_, ranged from 0.28–0.95. As the change in EELV is ΔPEEP × C_L_, lung compliance can be determined as ΔEELV/ΔPEEP, without oesophageal pressure, by increasing PEEP and determine ΔEELV.
**Figure S10:** Recording of inspiratory and expiratory tidal volumes before any correction of the offset between them (**upper left panel**), where V_Ti_ is constantly higher than V_Te_ during steady state ventilation. In the **second left panel**, V_Te_ is equalized to V_Ti_ at baseline, which results in V_Te_ being higher than V_Ti_ at end of PEEP_1_ period. In the **third left panel**, V_Te_ at end of PEEP_1_ period is equalized to V_Ti_ again. In lower right panel, V_Te_ is corrected with the same factor as for PEEP_1_ and then extrapolated to 25 breaths, where V_Te_ is assumed to be equal to V_Ti_.
**Figure S11:** Measurement procedure and calculations. **Panel A:** PEEP and airway plateau pressure. **Panel B:** Inspiratory and expiratory tidal volume (V_Ti_, V_Te_), where the offset between V_Ti_ and V_Te_ at steady state has been corrected for at baseline, at PEEP_1_ and at PEEP_2_. **Panel C:** Breath‐by‐breath difference between V_Ti_ and V_Te_ (ΔEELV). **Panel D:** Cumulative ΔEELV calculated as the cumulative difference between V_Ti_ and V_Te_ between PEEP levels. **Panel E:** Tidal airway P/V curves positioned at respective PEEP level. **Panel F:** The best fit lung P/V curve connecting the end‐expiratory airway = transpulmonary pressure P/V points. The green arrows indicate pleural driving pressure (ΔP_PL_). **Panel G:** The complete lung P/V curve calculated from baseline PEEP to end‐inspiratory transpulmonary plateau pressure at PEEP_2_ (P_Lplat_). Estimated values in italics and dashed lines. Blue arrow: tidal lung P/V curve at optimal PEEP level. ΔVoa = lung volume increase from end‐expiration at baseline PEEP to end‐inspiratory ΔEELV of the two PEEP steps + a tidal volume. ΔP_oa_ = end‐inspiratory transpulmonary plateau pressure at PEEP_2_ minus baseline PEEP. C_Loa_ = overall lung compliance = ΔV_oa_/ΔP_oa_.
**Figure S12: Left panel:** The best fit lung P/V curve connecting the end‐expiratory airway = transpulmonary pressure P/V points. The end‐inspiratory transpulmonary plateau pressure at baseline and PEEP_1_ are calculated from the second‐degree equation for the best fit lung P/V curve. The green arrows indicate pleural driving pressure (ΔP_PL_).
**Figure S13:** Optimal PEEP level determined as the pressure level half a tidal volume below, and transpulmonary plateau pressure at optimal PEEP half a tidal volume above the steepest point of the third‐degree equation of the best fit lung curve between end‐expiration at baseline PEEP and transpulmonary plateau pressure at the highest PEEP level (PEEP_2_).
**Figure S14:** Final results on lung mechanics displayed by the software. CLoverall = lung compliance calculated as ΔEELV_1_ + ΔEELV_2_ + V_T_/(P_Lplat_ of PEEP_2_—PEEP at baseline), V_Ti_ = inspiratory tidal volume, PEEP = end‐expiratory airway pressure, P_Lplat_ = end‐inspiratory transpulmonary plateau pressure at PEEP_2_, ΔP_AW_ = airway driving pressure, DP_L_ = transpulmonary driving pressure. Red P/V curves = tidal airway P/V curves, Thin blue line = lung P/V curve from end‐expiration at baseline PEEP to end‐inspiratory transpulmonary plateau pressure at PEEP_2_. Thick red P/V curve = tidal airway P/V curve at optimal PEEP, Thick blue P/V curve = tidal transpulmonary P/V curve at optimal PEEP.

## Data Availability

The data that support the findings of this study are available from the corresponding author upon reasonable request and the PSM method is described in detail in e‐supplement.
