# Supplementary material for: Inter‐Individual Variation in LUNG Mechanics Assessed by the Non‐Invasive PEEP‐Step Method During Laparoscopic and Robotic Surgery Indicates Need for Individualized Ventilatory Settings
Source: Acta Anaesthesiol Scand. 2026 Nov 5;71(1):e70388. doi: 10.1111/aas.70388 (PMC13739605; doi:10.1111/aas.70388)
Supplement: Supplementary file 1 [file AAS-71-0-s001.docx]

# **E-supplement of**

**Inter-individual variation in LUNG mechanics assessed by the non-invasive PEEP-step method during laparoscopic and robotic surgery indicates need for individualized ventilatory settings.**

Ali Abdulhussein^1^, Sara Hammarskjöld^2^, Ola Stenqvist^1^, Per Persson^1^, Katarina Hallén^1^, Pether Jildenstål^1^, Sophie Lindgren^1^*

Running title: **Lung mechanics in laparoscopic surgery**

## *Corresponding author: [sophie.lindgren@vgregion.se](mailto:ali.abdulhussein@vgregion.se)

1) Sahlgrenska University Hospital, Institute of Clinical Sciences, Gothenburg, Sweden
2) University of Gothenburg, Sahlgrenska Academy, Gothenburg, Sweden

**Page 2-6	Results of the present study**

**Page 7 – 15	Description of PEEP step method**

**Background physiology**

**Determination of ΔEELV**

**Measurement procedure**

**Calculation of lung P/V curve**

**Calculation of PEEP**

**Page 16	References**

**Present study**

All included patients were anaesthetised, intubated and ventilated according to standard clinical procedure by the attending anaesthesiologist. General anaesthesia was induced with propofol 2 mg ·kg ^-1^ (Propolipid Fresenius Kabi, Germany), fentanyl 2,5 µgram· kg^-1^ (Braun, Germany) and rocuronium 0.6 mg ·kg^-1^ (Fresenius Kabi, Germany) and maintained with sevoflurane 0.3-0.5 MAC (AbbVie, Sweden), remifentanil 0,20-0,40 μg ·kg^-1^ ·min^-1^ (Braun, Germany) and rocuronium 0,15 mg ·kg^-1^ iterated.

Lung compliance (C_L_) increased more than 30 %, from 98 ± 27 ml/cmH_2_O to 131 ± 32 ml/cmH_2_O, when PEEP was increased from baseline level of 6.5 ± 1.8 cmH_2_O to 11.8 ± 2.2 cmH_2_O at PEEP_1_ during the measurement procedure after induction. At the highest PEEP level C_L_ decreased as a sign of beginning of overstretching +++. During the measurement procedure after CO_2_+tilt and CO_2_, C_L_ continued to increase also at the highest PEEP level (PEEP_2_), which indicates that there was no overstretching of the lung but rather a lung that was compressed from the chest wall complex (Fig. S1).

***Figure S1.*** *Lung compliance for the patients at baseline PEEP, PEEP_1_ and PEEP_2_ during the measurement procedure after induction (left panel), after CO_2_/CO_2_+tilt (mid panel) and before wake-up (right panel). The black dashed line indicates mean values of C_L_.*

Optimal PEEP was 12.8 ± 2.7 cmH_2_O, ranging from 5.0 to 19.7 cmH_2_O and unrelated to lung compliance at baseline PEEP direct after induction. Lung compliance of optimal PEEP was average 30 % higher than lung compliance at baseline PEEP after induction with great individual variation (fig. S2, S3, S4, S5, S6).

***Figure S2.*** *Optimal PEEP calculated by equation for lung P/V curve at induction.*

***Figure S3.*** *Lung compliance at optimal PEEP compared to lung compliance at baseline PEEP at induction.*

***Figure S4.*** *Transpulmonary driving pressure at baseline PEEP (blue bars) and at optimal PEEP (brown bars) at induction.*

***Figure S5.*** *Optimal PEEP, Transpulmonary driving pressure and transpulmonary plateau pressure at optimal PEEP. Blue, black and brown dashed lines indicate mean optimal PEEP, ΔP_L_ and P_Lplat_ of optimal PEEP. Red thin dashed line indicate upper safety level of transpulmonary plateau pressure[1] and red thick dashed line indicates upper safety level for transpulmonary driving pressure [2-4].*

*.*

***Figure S6.*** *ΔEELV at optimal PEEP after induction. Patients are ranked after C_L_ at baseline PEEP*

Lung compliance at baseline PEEP was generally higher in the CO_2_+tilt group than in the CO_2_+supine group at induction which probably was an effect of PEEP being significantly higher in the CO_2_+tilt group. The effect of CO_2_ and CO_2_+tilt on C_L_ varied greatly between patients and was more pronounced in the CO_2_+tilt group (Fig. S7).

***Figure S7.*** *Upper panels: C_L_ at baseline PEEP at induction, CO_2_+tilt (left panel) and CO_2_ supine (right panel) and at wake up. Lower panels: ΔP_L_ at baseline PEEP at induction, CO_2_+tilt (left panel) and CO_2_ supine (right panel) and at wake up. Transpulmonary driving pressure was generally normal-low at induction.*

In most figures patients are ranked from lowest to highest lung compliance at baseline PEEP direct after induction. However, the response to increasing PEEP is highly variable, and a high C_L_ at baseline can change significantly when increasing PEEP, as seen during the two PEEP step measurement procedure. In the figure below (Fig. S8), every fourth patient best fit lung P/V curve by the two PEEP step procedure is displayed ranked by C_L_ at baseline PEEP. The curvature of the lung P/V curve cannot be predicted from C_L_ at baseline PEEP.

***Figure S8.*** *Lung P/V curves of each fourth patients ranked after C_L_ at baseline PEEP direct after induction. Note that the response to a PEEP step increase is highly variable. Overall lung compliance (C_Loa_) is the two-point compliance between end-inspiratory lung volume at PEEP_2_ divided by end-inspiratory transpulmonary plateau pressure at PEEP_2_ minus PEEP at baseline. The thick blue arrow is the tidal lung P/V curve at optimal PEEP, i.e., the PEEP level where the transpulmonary driving pressure is lowest (least injurious).*

**The PEEP step method**

Transpulmonary pressure increases in relation to volume inflated and the elastic properties of the lung, ΔP_L_ = ΔV/C_L_, irrespective of whether the increase in volume is caused by a conventional tidal inflation or by an increase in PEEP. The airway driving pressure (ΔP_AW_) is the combined pressure of needed to overcome the elastic recoil of the lung and to push the chest wall complex (chest wall, diaphragm and abdomen) away. To find out how much of ΔP_AW_ that represents ΔP_L_, conventionally oesophageal pressure measurements are used to determine the driving pressure of the chest wall complex (ΔP_ES_), which then can be subtracted from airway driving pressure to obtain ΔP_L,_ = ΔP_AW_ - ΔP_ES_.

As the chest wall strives outwards to its resting volume at 70-80 % of total lung capacity, it does not lean on the lung at end-expiration if the lung volume is below that level. That means that PEEP causes a breath-by-breath end-expiratory inflation of the lung as PEEP inflation is not impeded by the chest wall complex. The end-expiratory airway pressure of the ventilator only has to overcome the elasticity of the lung, while the expansive chest wall, i.e., the rib cage spring out force moves the chest wall outwards in parallel with the inflation of the lung. This is confirmed by comparison of change in end-expiratory lung volume (ΔEELV) measured by the ventilator pneumotachograph with ΔEELV calculated as ΔPEEP x C_L_, where C_L_ was determined as the tidal volume (VT) divided by the difference in airway and oesophageal driving pressure, C_L_ = V_T_/(ΔP_AW_-ΔP_ES_) (Fig. 1). A comparison of cumulative PEEP with the cumulative increase in end-expiratory transpulmonary pressure (ΔP_LEE_), confirmed that ΔP_LEE_ increases as much as PEEP is increased (Fig. S9)[5].

As the change in EELV is ΔPEEP x C_L_, lung compliance can be determined as ΔEELV/ΔPEEP, without oesophageal pressure, by increasing PEEP and determine ΔEELV.

Thus,

C_L_ = ΔEELV/ΔPEEP and

C_L_ = V_T_/(ΔP_AW_ – ΔP_ES_) = V_T_/ΔP_L_

and consequently, when ΔEELV = V_T_, i.e. when a change in PEEP from baseline PEEP level causes an increase in ΔEELV that is equal to the tidal volume from baseline PEEP

ΔPEEP = ΔP_L_

which implies that at a certain lung volume level, transpulmonary pressure is the same, irrespective of whether this volume has been reached by tidal or PEEP inflation.

The PEEP step method has been validated, described and commented on in several articles over the years: [1, 5-7, 11-21]

***Figure S9.*** ***Left panel:*** *Correlation plot of cumulative ΔEELV measured PEEP step by PEEP step by the ventilator pneumotachograph versus cumulative ΔEELV calculated PEEP step by PEEP step as ΔPEEP x C_L_ (E_L_ = 1/C_L_) in Katz I [6], Katz II [7], Falke [8], Persson [9], Lundin [10], Stenqvist [11], Garnero [12], Pelosi [13], Gattinoni [14]. E_L_ (= 1/C_L_) in these studies ranged from 9 – 43 cmH_2_O/L (C_L_: 111 – 23 ml/cmH_2_O) and the ratio of lung elastance to respiratory system elastance (E_RS_ = 1/C_RS_), E_L_/E_RS_ = ΔP_L_/ΔP_AW_, ranged from 0.28 – 0.95. As the change in EELV is ΔPEEP x C_L_, lung compliance can be determined as ΔEELV/ΔPEEP, without oesophageal pressure, by increasing PEEP and determine ΔEELV.*

***Right panel:*** *Correlation plot between cumulative ΔPEEP and cumulative ΔP_LEE_ calculated as ΔEELV/C_L_, PEEP step by PEEP step in [6-14], showing that P_LEE_ increases as much as PEEP is increased. The transpulmonary driving pressure (ΔP_L_) of a tidal volume equal to ΔEELV is equal to ΔPEEP. (With permission from Crit Care [5]).*

**Determination of ΔEELV** ΔEELV is determined as the cumulative difference between inspiratory and expiratory tidal volume (V_Ti_, V_Te_) between two PEEP levels (Fig. S9). Normally, there is an offset between V_Ti_ and V_Te_, which has to be corrected by equalizing V_Te_ to V_Te_ at steady state at baseline. When increasing PEEP, the offset changes and is corrected by assuming that between 20-25 breaths after the increase, V_Te_ should be equal to VTi. The same correction factor is used for the second PEEP step, which in the software normally is only around 12-14 breaths. The volume increase of this PEEP step is extrapolated to 25 breaths assuming that VTe should be equal to V_Ti_ after 25 breaths. The increase in EELV between breaths 12-14 till breath 25 is assumed occurring by 2/3 after half of the breaths 12-14 till breath 25 (Fig.S10).

***Figure S10****. Recording of inspiratory and expiratory tidal volumes before any correction of the offset between them (****upper left panel****), where V_Ti_ is constantly higher than V_Te_ during steady state ventilation. In the* ***second left panel****, V_Te_ is equalized to V_Ti_ at baseline, which results in V_Te_ being higher than V_Ti_ at end of PEEP_1_ period. In the* ***third left panel****, VTe at end of PEEP1 period is equalized to VTi again. In lower right panel, V_Te_ is corrected with the same factor as for PEEP_1_ and then extrapolated to 25 breaths, where V_Te_ is assumed to be qual to V_Ti_.*

*In the corresponding right panels, cumulative ΔEELV based on non-corrected offset between V_Ti_ and V_Te_ (****upper right panel****). As the offset is around 30 ml/breath, cumulative ΔEELV shows an increase of 600 ml during 20 breaths during steady state ventilation.* ***Second right panel:*** *When V_Te_ is equalized to V_Ti_ during baseline, cumulative ΔEELV is constant, but the change in offset when PEEP is increased causes cumulative ΔEELV during PEEP_1_ to decrease after a peak five breaths after increasing PEEP).* ***Third right panel****: Equalization of V_Ti_ during the last breaths of PEEP_1_ period results in a “correct” ΔEELV build up.* ***Lower right panel:*** *As the second PEEP step is of the same size as the first, the offset is corrected by the same factor as for the first PEEP step. Finally, cumulative ΔEELV is extrapolated to 25 breaths, assuming that is the number of breaths involved in establishing a new steady state [6, 9].*

**Measurement procedure and calculation of lung P/V curve**

The software guided the researcher through the measurement procedure. As ΔP_L_ is on average 70% of ΔP_AW_ [12-15], and ΔP_L_ of a tidal volume equal to ΔEELV is equal to ΔPEEP, PEEP was increased by a default value of 70 % of ΔP_AW_ at baseline PEEP in two steps, with the aim to increase EELV within ± 20 % of the tidal volume. The software accessed end-expiratory and end-inspiratory airway pressures and inspiratory and expiratory tidal volumes breath by breath during the measurement procedure and then calculated ΔEELV as described above and the lung pressure/volume (P/V) curve and optimal PEEP as described below (Fig.S11 A-G):

1. PEEP was increased in two steps by a default value of 70% of the ΔP_AW_. The increase in PEEP could be higher or lower than the default recommendation increase, for clinical reasons, without jeopardizing measurements. 2. ΔEELV between baseline PEEP and PEEP_1_ as well as between PEEP_1_ and PEEP_2_ was determined as the cumulative difference in inspiratory and expiratory tidal volume between PEEP levels after correcting for changes in V_Ti_-V_Te_ off-set at each PEEP level (Fig. S11 B, C, D, 3) 3. A best fit second-degree lung P/V curve was calculated between the three end-expiratory airway pressure/EELV points (Fig. S11 D). 4. The end-inspiratory transpulmonary plateau pressure at baseline PEEP and PEEP_1_ was determined by calculating the volume at steps of 0.1 cmH_2_O from the equation for the best fit curve to find the pressure where the calculated volume was equal to the end-inspiratory plateau pressure at baseline PEEP and PEEP_1_ (Fig. S11 F, S12).

5. The end-inspiratory transpulmonary plateau pressure (P_LplatPEEP2_) at PEEP_2_ was estimated as the end-inspiratory airway plateau pressure at PEEP_2_ minus the mean of ΔP_PL_ at baseline and PEEP_1_ (Fig. S11 G). When ΔEELV_1_ + ΔEELV_2_ was considerably lower than ΔEELV_1_+VT, P_LplatPEEP2_ at PEEP_2_ was estimated as the end-inspiratory airway plateau pressure at PEEP_2_ minus ΔP_PL_ at baseline. 6. Optimal PEEP was determined as the PEEP level half a tidal volume below the steepest point of the lung P/V curve [7, 10] (Fig. S11 G, S13). 7. Overall lung compliance (CLoa) was calculated as (ΔEELV_1_ + ΔEELV_2_ + V_T_)/(P_LplatPEEP2_ – PEEP_BL_)

***Figure S11.*** *Measurement procedure and calculations.* ***Panel A:*** *PEEP and airway plateau pressure.* ***Panel B:*** *Inspiratory and expiratory tidal volume (V_Ti_, V_Te_), where the offset between V_Ti_ and V_Te_ at steady state has been corrected for at baseline, at PEEP_1_ and at PEEP_2_.* ***Panel C:*** *Breath-by-breath difference between V_Ti_ and V_Te_ (ΔEELV).* ***Panel D:*** *Cumulative ΔEELV calculated as the cumulative difference between V_Ti_ and V_Te_ between PEEP levels.* ***Panel E:*** *Tidal airway P/V curves positioned at respective PEEP level.* ***Panel F:*** *The best fit lung P/V curve connecting the end-expiratory airway = transpulmonary pressure P/V points. The green arrows indicate pleural driving pressure (ΔP_PL_).* ***Panel G:*** *The complete lung P/V curve calculated from baseline PEEP to end-inspiratory transpulmonary plateau pressure at PEEP_2_ (P_Lplat_) Estimated values in italics and dashed lines. Blue arrow: Tidal lung P/V curve at optimal PEEP level. ΔVoa = lung volume increase from end-expiration at baseline PEEP to end-inspiratory ΔEELV of the two PEEP steps + a tidal volume. ΔP_oa_ = end-inspiratory transpulmonary plateau pressure at PEEP 2 minus baseline PEEP. C_Loa_ = overall lung compliance = ΔV_oa_/ΔP_oa_.*

**Calculation of the lung P/V curve**

It is an inherent feature of the PEEP step method that end-inspiratory transpulmonary plateau pressure at the highest PEEP level (PEEP_2_) cannot be measured but has to be estimated. Estimation of the transpulmonary plateau pressure at the highest PEEP level in the software is based on extrapolation of ΔP_PL_ at baseline and PEEP_1_ to PEEP_2_ level. However, in the present study, where results were hand-calculated, ΔP_PL_ at PEEP_2_ level was determined as the mean of ΔP_PL_ at baseline and PEEP_1_, as in a post-hoc analysis of data from Persson et al, 2018 [9], the mean ΔP_PL_ as base of calculating the transpulmonary plateau at the highest PEEP level, was superior in identifying whether lung compliance increased, remained mainly constant or decreased at the highest PEEP level. The study included 24 patients, but one patient was excluded as it showed a reverse sigmoid lung P/V curve based on oesophageal pressure measurements. In the other 23 patients, lung compliance showed the same decrease, equal or increase pattern when transpulmonary pressure was determined by oesophageal pressure and when estimated as the airway plateau pressure at the highest PEEP level minus the mean “pleural“ driving pressure of baseline and PEEP_1_ level (Fig. S11, S12).

***Figure S12. Left panel:*** *The best fit lung P/V curve connecting the end-expiratory airway = transpulmonary pressure P/V points. The end-inspiratory transpulmonary plateau pressure at baseline and PEEP_1_ are calculated from the second-degree equation for the best fit lung P/V curve. The green arrows indicate pleural driving pressure (ΔP_PL_).*

The transpulmonary plateau pressure (P_Lplat_) at baseline PEEP and PEEP_1_ was determined by calculating the volume for each step of 0.1 cmH_2_O using the second-degree equation of the best fit lung P/V curve between the three end-expiratory airway P/V points = transpulmonary P/V points (Fig. S12 left panel). The P_Lplat_ at baseline PEEP is the pressure where the volume is equal to the tidal volume and the P_Lplat_ at PEEP_1_ is the pressure where the volume is equal to ΔEELV_1_+V_T_ (Fig. 11 F, S12).

**Optimal PEEP calculation**

Optimal PEEP was determined by calculating the volume for each step of 0.1 cmH_2_O using the third-degree equation of the best fit lung P/V curve from end-expiration at baseline to end-inspiration at the highest PEEP level (Fig. 13). The stepwise volume change (ΔV)between PEEP steps of 0.1 cmH_2_O was calculated and the pressure level where ΔV was highest indicates the steepest point of the lung P/V curve. The pressure half a tidal volume below is optimal PEEP level and the pressure half a tidal volume higher is the transpulmonary plateau pressure. The difference between these pressures is the transpulmonary driving pressure of the tidal volume at optimal PEEP (fig. S13)

After the measurement procedure, data on C_L_, optimal PEEP, ΔP_AW_, ΔPL and ΔP_L_/ΔP_AW_ were presented. The results were observed by the researcher but not used to guide the clinical ventilatory management to prevent bias.

***Figure S13.*** *Optimal PEEP level determined as the pressure level half a tidal volume below, and transpulmonary plateau pressure at optimal PEEP half a tidal volume above the steepest point of the third-degree equation of the best fit lung curve between end-expiration at baseline PEEP and transpulmonary plateau pressure at the highest PEEP level (PEEP_2_).*

After finishing the measurement procedure by returning PEEP to baseline level, the software reported results with focus on optimal PEEP data (fig. S14).

***Figure S14.*** *Final results on lung mechanics displayed by the software. CLoverall = lung compliance calculated as* Δ*EELV1 + ΔEELV_2_+V_T_/(P_Lplat_ of PEEP_2_ – PEEP at baseline), V_Ti_ = inspiratory tidal volume, PEEP = end-expiratory airway pressure, P_Lplat_ = end-inspiratory transpulmonary plateau pressure at PEEP_2_, ΔP_AW_ = airway driving pressure, DP_L_ = transpulmonary driving pressure. Red P/V curves = tidal airway P/V curves, Thin blue line = lung P/V curve from end-expiration at baseline PEEP to end-inspiratory transpulmonary plateau pressure at PEEP_2_. Thick red P/V curve = tidal airway P/V curve at optimal PEEP, Thick blue P/V curve = tidal transpulmonary P/V curve at optimal PEEP.*

**References**

1.	Grasso S, Terragni P, Birocco A, Urbino R, Del Sorbo L, Filippini C, Mascia L, Pesenti A, Zangrillo A, Gattinoni L *et al*: **ECMO criteria for influenza A (H1N1)-associated ARDS: role of transpulmonary pressure**. *Intensive care medicine* 2012, **38**(3):395-403.

2.	Loring SH, Malhotra A: **Driving pressure and respiratory mechanics in ARDS**. *The New England journal of medicine* 2015, **372**(8):776-777.

3.	Amato MB, Meade MO, Slutsky AS, Brochard L, Costa EL, Schoenfeld DA, Stewart TE, Briel M, Talmor D, Mercat A *et al*: **Driving pressure and survival in the acute respiratory distress syndrome**. *The New England journal of medicine* 2015, **372**(8):747-755.

4.	Persson P, Stenqvist O: **Protective positive end-expiratory pressure and tidal volume adapted to lung compliance determined by a rapid positive end-expiratory pressure-step procedure in the operating theatre: a post hoc analysis**. *British journal of anaesthesia* 2022, **128**(4):e284-e286.

5.	Stenqvist O: **Transpulmonary pressure monitoring in critically ill patients: pros and cons-correction of description of the non-invasive PEEP-step method for separation of lung and chest wall mechanics**. *Critical care* 2024, **28**(1):355.

6.	Katz JA, Ozanne GM, Zinn SE, Fairley HB: **Time course and mechanisms of lung-volume increase with PEEP in acute pulmonary failure**. *Anesthesiology* 1981, **54**(1):9-16.

7.	Katz JA, Zinn SE, Ozanne GM, Fairley HB: **Pulmonary, chest wall, and lung-thorax elastances in acute respiratory failure**. *Chest* 1981, **80**(3):304-311.

8.	Falke KJ, Pontoppidan H, Kumar A, Leith DE, Geffin B, Laver MB: **Ventilation with end-expiratory pressure in acute lung disease**. *The Journal of clinical investigation* 1972, **51**(9):2315-2323.

9.	Persson P, Stenqvist O, Lundin S: **Evaluation of lung and chest wall mechanics during anaesthesia using the PEEP-step method**. *British journal of anaesthesia* 2018, **120**(4):860-867.

10.	Lundin S, Grivans C, Stenqvist O: **Transpulmonary pressure and lung elastance can be estimated by a PEEP-step manoeuvre**. *Acta anaesthesiologica Scandinavica* 2015, **59**(2):185-196.

11.	Stenqvist O, Grivans C, Andersson B, Lundin S: **Lung elastance and transpulmonary pressure can be determined without using oesophageal pressure measurements**. *Acta anaesthesiologica Scandinavica* 2012, **56**(6):738-747.

12.	Garnero A, Tuxen D, Ducros L, Demory D, Donati SY, Durand-Gasselin J, Cooper J, Hodgson C, Arnal JM: **Non-invasive assessment of lung elastance in patients with acute respiratory distress syndrome**. *Minerva anestesiologica* 2015, **81**(10):1096-1104.

13.	Pelosi P, Cereda M, Foti G, Giacomini M, Pesenti A: **Alterations of lung and chest wall mechanics in patients with acute lung injury: effects of positive end-expiratory pressure**. *American journal of respiratory and critical care medicine* 1995, **152**(2):531-537.

14.	Gattinoni L, Pelosi P, Suter PM, Pedoto A, Vercesi P, Lissoni A: **Acute respiratory distress syndrome caused by pulmonary and extrapulmonary disease. Different syndromes?** *American journal of respiratory and critical care medicine* 1998, **158**(1):3-11.

15.	Chiumello D, Carlesso E, Cadringher P, Caironi P, Valenza F, Polli F, Tallarini F, Cozzi P, Cressoni M, Colombo A *et al*: **Lung stress and strain during mechanical ventilation for acute respiratory distress syndrome**. *American journal of respiratory and critical care medicine* 2008, **178**(4):346-355.
